# A descriptive cost comparison of Listening and Spoken Language – South Africa versus traditional Speech-Language Therapy as therapeutic approaches for deaf and hard-of-hearing children in South Africa

**DOI:** 10.4102/jphia.v17i1.1735

**Published:** 2026-07-09

**Authors:** Aisha Casoojee, Katijah Khoza-Shangase

**Affiliations:** 1 Department of Audiology, Faculty of Humanities, University of the Witwatersrand, Johannesburg, South Africa

**Keywords:** cost analysis, early intervention, hearing impairment, South Africa, education policy, low-income and middle-income countries

## Abstract

**Background:**

Hearing impairment affects speech-language and academic development, creating substantial lifelong public health and economic burdens. In South Africa, early intervention typically follows either the Listening and Spoken Language–South Africa (LSL-SA) or Traditional Speech-Language Therapy (TSLT) model. Although LSL-SA demonstrates promising outcomes, limited evidence compares its costs with TSLT in low- and middle-income countries (LMICs). Such evidence is needed to inform equitable health financing and disability-inclusion policies.

**Aim:**

This study aimed to compare the total and component costs of LSL-SA and TSLT; describe cost distribution across healthcare, education and family domains and identify barriers to accessibility and scalability.

**Setting:**

This study was conducted in South Africa and examined intervention pathways for children with hearing impairment.

**Methods:**

A comparative costing approach was used to estimate expenditures across healthcare, education, and family sectors and compare the economic implications of LSL-SA and TSLT.

**Results:**

Listening and Spoken Language–South Africa incurred higher upfront therapy and rehabilitation costs but achieved superior outcomes, with 78% of children placed in mainstream schools compared with 42% in the TSLT group. The average total cost per child was ZAR 1.08 million for LSL-SA and ZAR 1.02 million for TSLT. Healthcare accounted for 65% of LSL-SA expenditure, whereas TSLT incurred higher education costs (35%) because of greater reliance on special schooling. Overall, LSL-SA demonstrated a more favourable cost-outcome profile despite its higher initial investment.

**Conclusion:**

Despite greater upfront investment, LSL-SA produced better educational outcomes and a more favourable cost-outcome profile than TSLT.

**Contribution:**

This study provides LMIC-specific economic evidence to inform resource allocation, disability-inclusive policies, and investment in early hearing intervention.

## Introduction

### Background

Hearing impairment is one of the most common childhood disabilities, significantly affecting speech-language acquisition, cognitive development, and academic achievement.^1^ Early intervention (EI) plays a pivotal role in mitigating these effects, enabling children with hearing loss to develop age-appropriate language skills and transition into mainstream educational settings.^2,3,4,5^ However, the effectiveness of therapeutic approaches can vary depending on multiple factors, including the methodology used, access to resources, parental involvement, and the socioeconomic context in which services are provided.^6,7,8^

In the South African context, children who are deaf or hard-of-hearing (DHH) access EI through one of two primary therapeutic approaches: (1) Listening and Spoken Language–South Africa (LSL-SA), an auditory-verbal therapy (AVT)-inspired approach that emphasises maximising residual hearing through amplification (hearing aids or cochlear implants [CIs]) and structured listening-based language development^9,10^; and (2) Traditional Speech-Language Therapy (TSLT), which incorporates multiple modalities, including speechreading, gestures, and limited sign support, focusing on both auditory and visual communication strategies.^11^ Both approaches have demonstrated positive outcomes in DHH populations^9^; however, their comparative value in terms of clinical and educational impact remains underexplored. Given South Africa’s resource constraints, healthcare disparities, and policy limitations in EI,^12,13,14^ it is critical to identify which model delivers the greatest return on investment within a resource-constrained health system. Understanding the economic and clinical implications of each approach is therefore essential for guiding equitable and sustainable early hearing care. Within a public health framework, early hearing intervention represents a high-yield investment. Unaddressed childhood hearing loss contributes to lifelong communication barriers, social exclusion, and reduced productivity, imposing a measurable burden on both the healthcare system and the broader economy.^1^ Evaluating intervention models through an economic lens supports rational priority-setting in resource-constrained contexts.

In this context, it is important to recognise that South Africa’s public healthcare system operates under an H0 policy framework, under which healthcare is provided free of charge at the point of service to children aged 0–6 years. This has major implications for how EI costs are distributed: while families do not directly bear these medical expenses, the Department of Health (DoH) absorbs these costs at a system level. However, system-level sunk costs (e.g. infrastructure and staffing) are typically excluded from cost analyses, which can lead to an underestimation of the true societal investment required.

There are significant cost considerations in EI for DHH children. Providing high-quality therapeutic services involves direct and indirect costs across multiple sectors, including healthcare, education, and family domains. Healthcare costs include medical, surgical and audiological services, hearing technology, therapy sessions, and habilitation. Educational costs include special education services, in-school support, and placement in mainstream or special schools. Family costs include transport, assistive devices, and lost income because of caregiver responsibilities.^4^ While LSL-SA is associated with better speech-language and educational outcomes,^2,6^ it requires early diagnosis, specialised clinician training, intensive and consistent parental involvement, and early access to hearing technology^9^ raising important questions regarding scalability and feasibility in under-resourced contexts such as South Africa. In contrast, TSLT, although more widely accessible in the public system, may involve longer therapy durations and increased reliance on specialised educational support, potentially leading to higher cumulative costs over time. In this context, clarifying the actual financial inputs associated with both intervention models is essential. Globally, cost analyses in high-income countries (HICs) have demonstrated that early cochlear implantation and intensive AVT can yield long-term cost savings through reduced reliance on special education and improved employment outcomes.^15,16^ However, comparable data from low-income and middle-income countries (LMICs) remain scarce, particularly within publicly financed health systems, highlighting the relevance of context-specific evidence from settings such as South Africa.

This study addresses a key gap by providing a descriptive cost comparison of LSL-SA and TSLT within an LMIC context. By integrating healthcare, educational, and family-level expenditure data, it offers a more comprehensive understanding of the true cost burden associated with EI pathways. To inform evidence-based policy and practice, there is a critical need to understand the economic implications of EI models for DHH children in South Africa. While future cost-effective analysis may provide more detailed insights into long-term value for investment, this study takes an essential first step. Specifically, the study seeks to:

- Compare the total costs associated with LSL-SA vs. TSLT, including therapy expenses, hearing technology, and educational support.
- Contextualise these costs in relation to reported speech-language and educational outcomes (as reported in previous studies) in the context of intervention costs.
- Examine systemic financial barriers to access, particularly in public sector healthcare and education settings.
- Provide policy-relevant insights for optimising funding and resource allocation.

While cost-effectiveness frameworks such as the Incremental Cost-Effectiveness Ratio (ICER), Quality-Adjusted Life Years (QALYs), and Disability-Adjusted Life Years (DALYs) provide useful models for evaluating intervention efficiency,^17,18^ this study does not apply these approaches. Instead, it focuses on presenting real-world cost data to highlight financial patterns that influence access, feasibility, and scalability of services. The findings are intended to inform healthcare financing, disability policy, and education planning for DHH children in South Africa. By demonstrating how intervention models with differing outcomes incur comparable costs, this study provides a foundation for policy decisions related to equitable resource allocations, EI prioritisation, and integrated service delivery. Ultimately, this study contributes novel empirical evidence to support public-sector planning and health-economic decision-making, with relevance for other LMICs facing similar resource constraints.

## Research methods and design

### Study design

This study employed a retrospective–prospective descriptive cost comparison design^19^ to examine the financial inputs associated with two intervention models for DHH children: (1) LSL-SA and (2) TSLT in South Africa. This approach enabled a structured comparison of direct and indirect costs across healthcare, education, and family domains, without inferring causality or conducting full economic evaluation modelling. This hybrid retrospective–prospective descriptive approach was selected because cost data spanning EI and primary schooling are rarely captured longitudinally in routine systems. Combining multiple data sources enhanced validity and approximated a societal perspective on cost distribution, which is consistent with World Health Organization (WHO) economic evaluation guidance.

All costs were calculated from birth to Grade 3, noting that the 0–6 years period is covered under South Africa’s free public healthcare policy (H0 policy). Direct DoH costs were included; however, sunk system-level costs (e.g. hospital infrastructure and staff salaries) were excluded, as these are not routinely captured in patient-level costing analyses.

### Participants and data sources

This study employed secondary data analysis supplemented with prospective cost estimation. Participants were 126 DHH children diagnosed with severe to profound congenital or early-onset hearing loss who had received EI through either LSL-SA (*n* = 62) or TSLT (*n* = 64). The sample was drawn from urban and peri-urban provinces (Gauteng, Western Cape, KwaZulu-Natal, and Eastern Cape), representing varied service delivery contexts and socioeconomic strata. Data sources included clinical records (therapy duration, medical, surgical, and audiology costs, and aided hearing thresholds), educational records (school placement, literacy outcomes, and grade retention), parental surveys (financial burden and accessibility of interventions), and programme invoices and cost estimates. Importantly, this study did not conduct cost-effectiveness modelling (e.g. ICER or Monte Carlo simulations), but rather focused on descriptive cost comparisons and cost–outcome interpretation to inform policy-relevant insights.

#### Inclusion criteria

The inclusion criteria were as follows:

- Children diagnosed with bilateral severe to profound hearing loss.
- Enrolment in either LSL-SA or TSLT intervention programmes before the age of 3 years.
- Minimum of 2 years of therapy completion with documented outcome measures.
- Available records on therapy duration, school placement, and speech-language scores.

#### Exclusion criteria

The exclusion criteria were as follows:

- Children with additional neurodevelopmental disorders (e.g. autism, cerebral palsy) that may confound language outcomes.
- Participants with incomplete therapy or school records.
- Families who did not consent to the use of cost data.

#### Sample selection

A matched-group comparative design^20^ was applied to ensure comparability between intervention groups. The LSL-SA group (*n* = 62) comprised children who received LSL-SA-based auditory-verbal intervention, while the TSLT Group (*n* = 64) comprised children who received TSLT-based multimodal therapy. Matching was conducted based on age at intervention, degree of hearing loss, socioeconomic background, and school type to minimise selection bias. This approach strengthened internal validity and improved comparability of cost and outcome patterns between groups.

Data sources included:

- Clinical records: Audiology (device type and follow-up sessions, aided thresholds) and speech-language therapy (communication outcomes data, duration of therapy attendance, sessions) (from the year 2008–2019).
- Educational records: School placement (mainstream vs. special school), grade retention, and academic performance summaries.
- Parent surveys: Structured questionnaires to gather information on out-of-pocket costs (transport, device maintenance, indirect costs).
- Private and public sector reference data: Published or publicly available cost estimates for hearing aids, CIs, therapy sessions, healthcare, and educational services.

### Data collection

#### Cost data collection

To estimate the full cost of intervention per child, both direct and indirect costs were considered across healthcare, education, and family domains (Table 1). All cost data were recorded in South African Rand (ZAR) and standardised to 2025 values to ensure comparability across time.

**TABLE 1:** Direct and indirect cost components collected.

Cost component	Data source	Unit of measurement
Audiology services	Clinic records	Cost per visit, total number of visits
Medical and surgical services	Parent-reported + billing data	Cost per procedure or session
Speech therapy sessions	Billing data	Cost per session, total number of sessions
Hearing technology	Parent-reported + clinic data	Device Purchase (hearing aids and/or cochlear implants), repairs, maintenance, and insurance
Educational support	School records	Tuition Fees (special school vs. mainstream school)
Parental costs	Questionnaire	Transport costs, lost wages because of caregiving

vs., versus.

To enhance accuracy, cost data were triangulated across multiple sources, including hospital billing systems, mainstream and special education cost reports, and insurance reimbursement records. Where data discrepancies occurred (e.g. parent reports vs. billing), mean values were cross-validated against institutional pricing schedules and published benchmarks to enhance reliability.

#### Costing assumptions

Several key assumptions guided the development of the costing framework in this study. Costs were estimated from both health system and family perspectives. Device-related costs were assumed to be borne by the DoH, while out-of-pocket costs were captured at the household level. For children under 6 years, healthcare services were provided under the H0 policy; therefore, no direct user fees were included for this period. The costing window extended from intervention onset to the end of Grade 3, consistent with the study’s educational outcome horizon. All assumptions were applied consistently across both groups to ensure comparability. Cost ranges were assumed to be uniformly distributed, and all values were standardised to ZAR (2025). Cochlear implantation was costed per procedure.

#### Outcome data collection

Intervention outcomes were assessed using speech-language and academic indicators derived from therapy and school records (Table 2).

**TABLE 2:** Outcome data collected, sources and criteria.

Outcome measure	Data source	Scoring criteria
Speech intelligibility	Therapy records	% intelligible speech (rating scale)
Expressive and receptive language	Standardised tests	Language percentile scores
Mainstream school placement	School records	Yes or no
Academic performance	Grade reports	Math and literacy scores

The contextualised cost findings and outcomes were used descriptively to interpret functional and educational gains associated with each intervention.

Educational performance was further synthesised into a ‘mainstream readiness’ indicator, derived from literacy, numeracy, and school placement data, to facilitate comparison between cost and functional outcomes.

#### Data analysis

Cost data were summarised descriptively using means, ranges, and proportional distributions across cost domains. No inferential statistical testing or formal cost-effectiveness modelling was conducted. Instead, analysis focused on identifying patterns in cost distribution and linking these to observed educational outcomes to support a cost–outcome interpretation. Findings were analysed to identify key financial drivers, access barriers, and implications for health system planning and policy.

### Ethical considerations

Prior to the commencement of the study, ethical approval was obtained from the University of the Witwatersrand Human Research Ethics Committee (Non-Medical) (Protocol Number: H20/06/03). Parents provided written consent for the use of anonymised data. All records were de-identified and securely stored. Potential bias was minimised through matched-group design and careful data triangulation; however, residual confounding cannot be fully excluded given the observational nature of the study.

As this study involved retrospective analysis of existing clinical, educational, and cost data, as well as parent-reported information, children were not directly engaged in data collection processes. Therefore, child assent was not obtained. However, parental consent was secured for the use of anonymised data, and all records were de-identified prior to analysis to ensure confidentiality and protection of participant rights. This approach is consistent with ethical guidelines for secondary data analysis involving minors.

## Results

### An overview of cost profiles

Costs were calculated across four domains: (1) medical and surgical services, (2) hearing technology, (3) audiology and rehabilitation, and (4) education and parental indirect costs. Results are presented separately for private and public healthcare pathways, as well as for LSL-SA and TSLT. All costs were calculated up to the end of Grade 3. For children aged 0–6 years accessing public healthcare, services were free at the point of use under the H0 policy. Accordingly, direct DoH expenditures such as surgery, devices, and therapy services were included, while system-level sunk costs (e.g. infrastructure and staff salaries) were excluded. Overall, cost patterns differed substantially by sector, with private care characterised by high out-of-pocket expenditure and public care reflecting state-subsidised service provision.

It is important to note that formal school enrolment age in South Africa varies between 6 years and 7 years, depending on birth date, which may influence the timing of entry into Grade 1 and the duration of pre-primary education – where one excludes delayed enrolment because of challenges with finding an appropriate school, particularly for children with special needs. This introduces some variability in the distribution of early educational costs across participants. However, all costs were standardised to a common analytical endpoint (end of Grade 3), ensuring comparability across intervention groups despite these variations.

### Medical and surgical services

For families accessing private healthcare, CI surgery constituted a substantial initial cost, ranging from ZAR250 000 to ZAR300 000 per procedure. This included fees for Ear, Nose and Throat (ENT) Specialist fees, hospitalisation, and anaesthesia. In contrast, these costs were fully covered by the state in the public healthcare system, resulting in no direct out-of-pocket expenditure for families. Follow-up ENT visits and pre-operative consultations added further private costs but were similarly subsidised in the public sector (Table 3).

**TABLE 3:** A summary of medical and surgical costs (South African Rand, 2025 values).

Cost component	Private (LSL-SA and TSLT)	Public (LSL-SA and TSLT)
CI surgery (total)	250 000 – 300 000	Free
Pre-operative ENT (2–3 visits)	2000 – 4000 per visit	Free
ENT follow-up (×4 visits)	600 – 1800 per visit	Free

CI, Cochlear Implant; ENT, Ear Nose and Throat Specialist; LSL-SA, Listening and Spoken Language Intervention in South Africa; TSLT, Traditional Speech-Language Therapy.

Overall, Table 3 demonstrates that medical and surgical costs represent a major upfront financial burden in the private sector, whereas these costs are absorbed entirely by the public health system.

### Hearing technology

Hearing technology costs differed significantly between sectors. In the private sector, CI devices ranged from ZAR244 000 to ZAR306 000 per device, compared to ZAR70 000 – ZAR153 000 in the public sector. Hearing aid costs followed similar trends: private (ZAR10 810 – ZAR70 000) versus public (ZAR2500 – ZAR10 000). Frequency Modulation (FM) systems, which support classroom listening, added substantial costs in private care (ZAR36 000 – ZAR51 000) but were minimal in public settings (ZAR3400).

These findings (Table 4) indicate that hearing technology constitutes a major cost driver, particularly in private care, with significantly reduced costs achieved through public procurement systems.

**TABLE 4:** Hearing technology costs (South African Rand, 2025 values).

Cost component	Private	Public
CI device (per unit)	24 4000 – 306 000	70 000 – 153 000
Hearing aid (per unit)	10 810 – 70 000	2500 – 10 000
FM system	36 000 – 51 000	3400

CI, cochlear implant; FM, frequency modulation.

### Maintenance and upgrades

Ongoing maintenance and periodic upgrades added substantial long-term costs (Table 5). Cochlear implant upgrades were required every 3 years – 4 years, with higher costs in private care compared to public healthcare (ranging from ZAR180 000 to ZAR213 000 in private care compared to ZAR138 000–ZAR173 000 in the public system). Recurring expenses included disposable or rechargeable batteries, chargers, repairs, and device components.

**TABLE 5:** Key maintenance and upgrades costs (South African Rand, 2025 values).

Category	Component	Private	Public
CI maintenance	Maintenance plan (first 3 years)	ZAR10 000	Free
	Upgrade frequency	Every 3 – 4 years	Every 3 – 4 years
	Upgrade cost	ZAR180 718 – ZAR 213 503	ZAR138 000 –ZAR173 000
Power and battery costs	Repairs (10% probability)	10 200	9800
	Rechargeable batteries	4246 – 4399	3593 – 3737
	Disposable batteries (1–2 days)	102 – 119	97 – 108
Device accessories and spares	Coils	2123 – 4936	1898 – 2165
	Microphones	3668	3400
	Chargers	2909 – 7203	2385 – 4054
Hearing aid maintenance	Battery (10-day cycle)	20 – 27	20 – 27
	Upgrade cost	ZAR10 810 –ZAR70 000	ZAR2500 –ZAR10 000
Insurance costs (monthly premiums)	CI insurance	1016 – 1276	292 – 638
	HA insurance	45 – 292	10 – 42

CI, Cochlear Implant; HA, Hearing Aid; ZAR, South African Rand.

Maintenance and upgrade costs (Table 5) represent a substantial and ongoing financial burden across both sectors, particularly for CI users. While initial device acquisition is a major cost driver, recurring expenses related to batteries, repairs, and periodic upgrades contribute significantly to long-term cumulative costs. Among these components, device upgrades represent the largest single cost contributor, far exceeding routine expenses such as batteries and accessories. Although these costs are reduced in the public sector, they are not eliminated, particularly at the household level. While public-sector provision reduces upfront costs, the persistence of recurring expenses highlights that long-term device sustainability remains a shared financial burden across sectors.

### Audiology and rehabilitation

Audiology and rehabilitation services represented a major cumulative cost (Table 6), particularly in the private sector. Mapping sessions were most intensive in the first 2 years post-implantation (averaging 10 sessions in year 1, 5 in year 2, and then annually), with private costs ranging from ZAR600 to ZAR690 per session, with additional accommodation expenses incurred by ~10% of families. Mapping sessions refer specifically to CI programming visits, distinct from rehabilitation (therapy) sessions.

**TABLE 6:** Audiology and rehabilitation costs (South African Rand, 2025 values).

Category	Component	Private	Public
Audiology (CI and HA services)	Mapping sessions	600 – 690	Free
	CI audiology sessions	1200 – 1500	Free
	HA sessions	690 – 720	Free
Rehabilitation (therapy)	Duration	LSL-SA: 5.3 years; TSLT: 5.7 years	LSL-SA: 5.3 years; TSLT: 5.7 years
	Sessions/year	~44	~44
	Cost/session	ZAR320 – ZAR800	Free
Transport costs	Mode	Own car (100%)	Taxi (70%), car (30%)
	Cost/km	ZAR5.36	ZAR1.24 – ZAR5.36
	Distance	5 km – 45 km	5 km – 60 km

CI, Cochlear Implant; HA, Hearing Aid; LSL-SA, Listening and Spoken Language Intervention in South Africa; TSLT, Traditional Speech-Language Therapy; ZAR, South African Rand.

Rehabilitation duration averaged 5.3 years for LSL-SA and 5.7 years for TSLT, with approximately 44 sessions per year. Private therapy costs ranged from ZAR320 to ZAR800 per session, depending on duration, whereas services were provided free in the public sector.

Audiology and rehabilitation costs (Table 6) emerged as one of the most significant cumulative cost drivers, particularly in the private sector, where frequent therapy sessions are required over multiple years. While public sector provision eliminates direct service costs, indirect costs such as transport remain a substantial burden for families. Transport costs were estimated based on reported travel modes and average distance ranges.

### Educational costs

Education constituted a major long-term cost domain. In the private sector, 90% of children in both LSL-SA and TSLT groups attended private schools with annual tuition fees ranging from ZAR60 000 to ZAR110 400, alongside once-off entrance (ZAR150 – ZAR6000) and administrative fees (ZAR45 – ZAR500). In the public sector, children predominantly attended government schools, with annual fees substantially lower (ZAR15 000 – ZAR18 000) (Table 7). Importantly, children in the LSL-SA group were more likely to achieve mainstream school placement, reducing reliance on specialised schooling and associated long-term costs.

**TABLE 7:** Educational costs (South African Rand, 2025 values).

Category	Component	Private	Public
Educational costs (RR–Gr 3)	Years	5	5
	Annual fees	ZAR 60 000 – ZAR 110 400	ZAR15 000 – ZAR 18 000
	Entrance fees	ZAR150 – ZAR6000	ZAR150 – ZAR6000
	Admin fees	ZAR45 – ZAR500	ZAR45 – ZAR500
	Aftercare (monthly)	ZAR1940	ZAR1940
School type distribution	Private:	-	-
	LSL-SA	90%	90%
	TSLT	90%	90%
	Public:	-	-
	LSL-SA	10%	10%
	TSLT	10%	10%

LSL-SA, Listening and Spoken Language Intervention in South Africa; TSLT, Traditional Speech-Language Therapy; ZAR, South African Rand.

Educational costs constituted a major long-term expenditure domain. Importantly, differences in intervention outcomes influenced cost trajectories, with LSL-SA associated with higher rates of mainstream placement, thereby reducing reliance on specialised education and potential grade repetition. Aftercare costs were included as a monthly average where applicable, but may not apply to all households.

### Comparative cumulative costs

When aggregated (Table 8), cumulative per-child costs by Grade 3 exceeded ZAR1m in private care, driven by surgical, device, and schooling expenses. Public-sector costs were substantially lower because of state coverage of healthcare and rehabilitation services.

**TABLE 8:** A summary of cumulative cost drivers per child by sector (South African Rand, 2025 values).

Cost domain	Notes/Units	Private (ZAR)	Public (ZAR)
CI surgery	Per child	250 000 – 300 000	Free
Cochlear implant device	Per CI	244 000 – 306 000	70 000 – 153 000
FM system	Per system	36 000 – 51 000	3400
Rehabilitation sessions	5–6 years; per session	320 – 800	Free
CI upgrade	Per cycle	180 000 – 213 000	138 000 – 173 000
School fees	Annual	60 000 – 110 400	15 000 – 18 000
Parent transport	Cost per km	5.36 (own car)	1.24 (taxi)

CI, Cochlear Implant; HA, Hearing Aid; ZAR, South African Rand; FM, frequency modulation.

A clear shift in cost burden is observed across sectors: private-sector costs are front-loaded (surgery and devices), whereas public-sector costs are more distributed over time, with indirect household costs (e.g. transport) and educational expenses becoming more prominent.

Table 8 summarises the distribution of cost burden, illustrating the shift from healthcare-dominated costs in private care to education and transport costs in public care. Importantly, while public-sector costs appear substantially lower from a direct expenditure perspective, these estimates do not fully capture indirect household costs or system-level expenditures, suggesting that the true societal cost gap between sectors may be narrower than presented.

### Cost–outcome synthesis

When cost data are interpreted alongside educational outcomes, LSL-SA demonstrates a more favourable cost–outcome profile. Listening and Spoken Language–South Africa demonstrated a cost-per-mainstream-placement ratio of approximately ZAR1.38m per successfully mainstreamed child, versus ZAR2.43m for TSLT. Although it requires a higher upfront investment, it is associated with substantially higher mainstream school placement (78% vs. 42%), reducing reliance on specialised education and grade repetition. This suggests that early, intensive listening-based intervention may yield greater long-term efficiency, despite similar overall per-child costs between groups.

### Synthesis of cost and educational outcomes

To integrate cost findings with functional outcomes, a synthesis of cumulative expenditure and educational placement was undertaken. Although total per-child costs were broadly comparable between LSL-SA and TSLT by the end of Grade 3, the distribution and downstream implications of these costs differed substantially. Listening and Spoken Language–South Africa, despite higher upfront rehabilitation expenditure, was associated with significantly higher rates of mainstream school placement (78% vs. 42%), thereby reducing reliance on specialised education and mitigating long-term educational costs. In contrast, TSLT, while less intensive initially, demonstrated lower rates of mainstream integration, suggesting a shift of costs into the education sector over time. This pattern indicates that early, intensive auditory-verbal intervention may yield greater functional returns on investment, supporting a cost-efficiency advantage when educational outcomes are considered alongside direct financial inputs.

## Discussion

This study presents one of the first comparative cost analyses of intervention pathways for children with hearing loss in South Africa, comparing LSL-SA with TSLT across private and public healthcare settings. The findings demonstrate substantial differences in both the magnitude and distribution of costs, with private families shouldering significant direct financial burdens, while public-sector families primarily incurred indirect and educational expenses. From a health economics perspective, LSL-SA’s front-loaded investment aligns with the ‘pay now, save later’ principle typical of preventive interventions in public health. This pattern illustrates how early, intensive rehabilitation can offset downstream educational and social expenditures, akin to investments in immunisation and early childhood development in LMICs.

### Cost-effectiveness of interventions

The analysis highlights that while LSL-SA requires more intensive rehabilitation, it is associated with a higher likelihood of mainstream school placement. This carries important long-term economic implications, as mainstream schooling reduces reliance on costly special education, mitigates grade repetition, and supports social integration, ultimately improving relative cost-efficiency despite higher initial service use.^21,22,23^ This aligns with international evidence showing that investments in early, intensive rehabilitation yield substantial downstream savings through enhanced educational and employment outcomes.^15,16,24^

Although TSLT may appear less resource-intensive initially, delayed language outcomes and limited mainstream integration can shift costs downstream into special schooling and remedial education. These downstream costs perpetuate inequities in developmental outcomes. Therefore, LSL-SA emerges as a more cost-efficient strategy when considered from a lifetime perspective, even if total cumulative costs to Grade 3 are similar across groups. Importantly, the analysis accounted for variations in school enrolment age (6 years–7 years) and standardised costs to the end of Grade 3. This approach ensured comparability across children despite minor differences in start age, as highlighted in the Results and Methods section.

### Health equity considerations

The analysis exposes a profound equity gap between public and private pathways. In the private sector, out-of-pocket expenses frequently exceeded ZAR1m per child by Grade 3, an amount beyond the reach of most South African families. By contrast, the public system absorbed most surgical and device costs, yet parents still incurred transport, indirect care, and school-related expenses.

This duality reflects broader inequities in South African healthcare, where access to cochlear implantation and comprehensive rehabilitation is often mediated by socioeconomic status.^25^ While public provision offers critical financial relief, the burden of accessing services is shifted towards households, particularly through reliance on public or informal transport, high visit frequency, and associated time costs. Limitations in therapy availability, together with these indirect access barriers, may undermine continuity and intensity of rehabilitation. Ensuring that children in the public sector receive comparable intensity, consistency, and effectiveness in rehabilitation is therefore crucial. These inequities undermine commitments to Universal Health Coverage^1^ and UN Sustainable Development Goals (SDG),^26^ which emphasise early childhood development and disability inclusion.

Comparable patterns are observed in other LMICs such as Kenya, Nigeria, and Malawi, where cochlear implantation is primarily accessible through private or donor-funded programmes.^27^ The South African dual-system model provides a critical lens for continental policy dialogue on equitable access to auditory rehabilitation.

### Policy implications

The findings carry several actionable policy implications. Firstly, there is a compelling argument for expanding state-supported rehabilitation services, particularly intensive LSL-SA programmes, within public hospitals. Given the demonstrated long-term cost-efficiency of mainstream integration, investment in these services should be prioritised under the National Health Insurance (NHI) framework. Inclusion of paediatric hearing services within the essential benefits package is pivotal.^28^ Secondly, standardising costs and ensuring transparent procurement across provinces is essential. Wide variation in device and upgrade costs presents opportunities for bulk purchasing and tender negotiations to reduce national expenditure. Incorporating assistive device maintenance and upgrades into essential health benefit packages would promote sustainability.

Thirdly, policies addressing hidden costs such as transport subsidies, school fee relief, and provision of batteries and upgrades are necessary to reduce the disproportionate burden on low-income families. Without these measures, families may experience ‘treatment fatigue’ and disengage from follow-up, undermining initial investment.

Finally, this study supports the broader agenda of inclusive education policy, emphasising that hearing interventions cannot be siloed within health but must be integrated with educational planning.^29^ Collaboration between the Departments of Health and Basic Education is critical to translate investment in CIs into functional educational gains. Practical interdepartmental mechanisms may include joint funding of early identification and habilitation services, cross-training community health workers for referral pathways, and integrated school-readiness monitoring linking the Departments of Health and Basic Education databases. These findings echo evidence from other LMICs, where the long-term economic benefits of cochlear implantation outweigh upfront costs, but sustainability depends on subsidy mechanisms for devices and rehabilitation.^27,30^ Integrating LSL-SA into the NHI essential package, alongside subsidies to cover hidden costs, would represent a high-yield investment in reducing disability, promoting inclusion, and maximising long-term societal returns.

These recommendations support an integrated approach that maximises long-term societal return, advances SDG 3 (Good Health and Well-being) and SDG 4 (Quality Education) and addresses equity concerns in LMIC settings.

### Limitations and future directions

While this study provides valuable insights, several limitations must be observed. The retrospective design and reliance on secondary data introduce potential variability in cost estimates. Additionally, cost analysis was limited to Grade 3 endpoint; future studies should extend to adolescence and adulthood to capture full educational and employment trajectories. Prospective cost-effectiveness studies incorporating QALYs or similar health-economic metrics would strengthen the evidence base for policy decision-making.

Variation in school enrolment age (6 years – 7 years) may influence early education costs, although costs were standardised to Grade 3 to maintain comparability. Sunk system costs (infrastructure, staff salaries) were excluded, likely underestimating the true resource investment from the health system. The sample primarily included children already engaged in formal EI programmes, which may overestimate access and underestimate costs for rural populations or those outside structured programmes. Future research should incorporate formal cost-effectiveness metrics such as QALYs or DALYs, consider longer-term societal costs, and evaluate strategies to reduce inequities between private and public pathways.

## Conclusion

In conclusion, LSL-SA is more resource-intensive during the early years and demonstrates superior long-term cost-effectiveness because of higher mainstream school integration and reduces reliance on special schooling. Addressing persistent inequities between private and public pathways through expanded public rehabilitation, cost subsidies, and intersectoral collaboration is essential to achieving sustainable, equitable outcomes for DHH children in South Africa and similar LMICs. Future analyses incorporating cost-utility measures such as QALYs and DALYs will further quantify the long-term value of LSL-SA under universal coverage scenarios. Strengthening the evidence–policy interface through economic evaluation is crucial for integrating hearing health within South Africa’s preventive and primary healthcare agenda.
